# COVID-19 pandemic in minor psychiatric disorders among mobile emergency service workers: cohort

**DOI:** 10.1590/0034-7167-2025-0098

**Published:** 2026-05-11

**Authors:** Natasha da Silva Indruczaki Guedes, Polla Victória Paim Rodrigues Finckler, Silviamar Camponogara, Keyla Cristiane do Nascimento, Juliana Petri Tavares, Daiane Dal Pai

**Affiliations:** I Universidade Federal do Rio Grande do Sul. Porto Alegre, Rio Grande do Sul, Brazil; II Universidade Federal de Santa Maria. Santa Maria, Rio Grande do Sul, Brazil; III Universidade Federal de Santa Catarina. Florianópolis, Santa Catarina, Brazil

**Keywords:** COVID-19, Emergency Medical Services, Occupational Health, Mental Disorders, Cumulative Trauma Disorders., COVID-19, Servicios Médicos de Urgencia, Salud Laboral, Trastornos Mentales, Trastornos de Traumas Acumulados.

## Abstract

**Objectives:**

to measure the effect of the COVID-19 pandemic on minor psychological disorders among Mobile Emergency Care Service workers.

**Methods:**

this was a retrospective and prospective cohort study conducted in a southern Brazilian capital. Fifty-two workers (88.2% sample size) were monitored before the pandemic and two years later, using the Self-Reporting Questionnaire. Relative risk was estimated using the generalized estimating equations model and multiple analysis of variance for repeated measures, considering p<0.05.

**Results:**

disorders increased from 32.7% (n=17) to 40.4% (n=21) after two years of the pandemic (effect 0.23; p=0.12), with an increased risk for females (RR=2.10; 95%CI 1.11-3.97), comorbidities (RR=2.33; 95%CI 1.13-4.08), medication use (RR=2.37; 95%CI 1.30-4.33), nursing technicians (RR=4.44; 95%CI 1.19-16.56), and nurses (RR=6.41; 95%CI 1.69-24.24). The disorders were associated with neck and low back pain, physical, social, and psychological harm (p<0.01).

**Conclusions:**

minor psychological disorders had a high prevalence, intensifying during the pandemic.

## RESUMEN

**Objetivos:**

medir el efecto de la pandemia de COVID-19 en trastornos psicológicos menores entre trabajadores del Servicio Móvil de Atención de Emergencias.

**Métodos:**

estudio de cohorte retrospectivo y prospectivo realizado en una capital del sur de Brasil. Se monitoreó a cincuenta y dos trabajadores (88,2% del tamaño de la muestra) antes de la pandemia y dos años después, mediante el Self-Reporting Questionnaire. El riesgo relativo se estimó mediante el modelo de ecuaciones de estimación generalizadas y análisis de varianza múltiple para medidas repetidas, considerando un valor de p<0,05.

**Resultados:**

los trastornos aumentaron del 32,7% (n=17) al 40,4% (n=21) tras dos años de pandemia (efecto 0,23; p=0,12), con mayor riesgo en mujeres (RR=2,10; IC95% 1,11-3,97), comorbilidades (RR=2,33; IC95% 1,13-4,08), uso de medicación (RR=2,37; IC95% 1,30-4,33), técnicos de enfermería (RR=4,44; IC95% 1,19-16,56) y enfermeras (RR=6,41; IC95% 1,69-24,24). Los trastornos se asociaron con dolor de cuello y espalda baja, daño físico, social y psicológico (p<0,01).

**Conclusiones:**

los trastornos psicológicos menores presentaron una prevalencia alta, intensificándose durante la pandemia.

## INTRODUCTION

The COVID-19 pandemic required a reorganization of the Brazilian healthcare system. In emergency care, the Mobile Emergency Care Service (In Portuguese, *Serviço de Atendimento Móvel de Urgência* - SAMU) underwent changes in institutional flows and protocols, in addition to a different service profile^(1)^. Furthermore, there was an increase in team response time, considering the recommendation to clean the ambulances between patient care and transport^(2)^.

SAMU is part of the Brazilian healthcare system’s Emergency Care Network as a mobile pre-hospital component, coordinating the flow and providing immediate, effective, and safe care to victims distress from various health problems. The service operates through the deployment of specialized basic and advanced support teams, focused on life-threatening situations requiring immediate intervention and rapid, safe transportation to referral services^(1)^.

During the COVID-19 pandemic, pre-hospital care (PHC) workers experienced fear, insecurity, and fear of contracting the disease and infecting family members^(1)^, among other feelings that impacted their professional experiences and work routines. The pandemic period exacerbated pre-existing characteristics of PHC work routines. Numerous factors may be related to the development of psychological distress among these workers, such as a lack of safety in the workplace, lack of personal protective equipment, and excessive working hours^(3)^.

The presence of stressors related to team-based emergency care has been described in the literature, highlighting situations of insomnia, depression and anxiety, as well as stress and post-traumatic stress disorder^(4,5)^. An Iranian study found that working to rescue individuals affected by COVID-19 and experiencing distress increased anxiety levels, leading to psychological stress in the workplace due to concerns about infection, fear of the spread of the disease, distress of patients and their families, and the harm caused by personal protective equipment on nurses^(6)^. International studies have identified a greater likelihood of psychological distress in healthcare workers working on the front lines, such as depression, anxiety, insomnia, and distress^(7,8)^.

In Brazil, no studies were identified that assessed the impacts on SAMU workers’ mental health before and after the emergence of the pandemic caused by COVID-19, which justifies carrying out the study, considering that the impacts of the health crisis must be measured in all spheres of healthcare.

### Study relevance

This article is the product of a dissertation entitled “*Impacto da pandemia da Covid-19 no trabalho e adoecimento dos profissionais do Serviço de Atendimento Móvel de Urgência*”^(9)^, deposited in the institutional repository (http://hdl.handle.net/10183/259608).

## OBJECTIVES

To measure the effect of the COVID-19 pandemic on minor psychological disorders (MPD) in SAMU workers.

## METHODS

### Ethical aspects

The national ethical guidelines governing research in health, humanities, and social sciences were followed, in accordance with Resolutions 466/2012 and 510/2016 of the Brazilian National Health Council. All participants gave their consent by signing the Informed Consent Form.

### Study design, period and site

This is a retrospective and prospective cohort study, with a quantitative approach, in accordance with STrengthening the Reporting of Observational studies in Epidemiology guidelines.

Data were collected in different periods, with collections from December 2019 to February 2020 (pre-pandemic) and after two years in March 2022 (during the pandemic).

The research was conducted at SAMU in Porto Alegre, in the state of Rio Grande do Sul, Brazil. SAMU covers 1,332,845 million inhabitants and a territory of 495,390 km^2(10)^, through 16 teams, three of which are dedicated to Advanced Life Support, while the others, containing 13 units, provide Basic Life Support.

### Population or sample; inclusion and exclusion criteria

The population consisted of 260 healthcare workers (nurses, drivers, physicians, and nursing technicians) who worked in emergency care.

The pre-pandemic sample consisted of 113 SAMU workers. In the second collection period, conducted two years after the pandemic, the workers who had participated in the first period were included again. The final sample consisted of 52 participants, while 61 were lost due to absences and deaths.

The power to test the minimum difference of 2 points in the mean of the differences of the Self-Reporting Questionnaire (SRQ-20) between the pre and post groups is 88.2%, considering a significance level of 5%, sample size equal to 52 individuals and standard deviation of the differences equal to 4.5 points^(11)^. The PSS Health tool was used in the online version.

All workers were invited to participate in the research, being selected by convenience, considering that they were working during the data collection periods.

Workers who worked in care activities at the service and had a minimum contract of two years were included. Those who were on vacation or away from work during data collection were excluded. The second phase of the study, conducted two years after the pandemic, included workers who remained in care and who had responded to the instruments in the first phase of the study.

### Study protocol

Data collection was performed using instruments containing sociodemographic, clinical, and occupational data, as well as the SRQ-20. To obtain clinical data, in addition to general health questions, the Standardized Nordic Questionnaire (SNQ) was administered. To obtain occupational data, the *Escala de Avaliação dos Danos Relacionados ao Trabalho* (EADRT) was administered.

MPD were screened using the SRQ-20. This instrument consists of 20 questions, scored from 0 to 1, and tracks non-psychotic symptoms such as irritability, memory lapses, insomnia, difficulty concentrating, and fatigue, without establishing a clinical diagnosis. Having seven or more positive responses was considered a positive outcome^(12)^. The SNQ analyzes musculoskeletal symptoms in occupational health^(13)^.

The EADRT is one of the scales that make up the Work and Illness Risk Inventory, assessing workers’ perceptions of workplace-related harm to their health. Each question is assigned a score ranging from 1 to 6. A score below 1.9 points indicates a more positive perception of harm, considering it tolerable; scores between 2 and 3 reflect a moderate to critical assessment; scores of 3.1 and 4 indicate moderate to frequent impacts, considered severe; while scores above 4.1 indicate a more negative assessment, associated with occupational illnesses^(14)^. In this study, to maximize differences between groups, classifications were reduced into two: with occupational disease (score ≥ 4.1); and with serious work-related situations (score ˂ 4).

Data were collected at SAMU databases, based on workers’ schedule and availability. Workers were invited in person to participate in both phases of the study. The instruments were administered by a trained research team. In some situations, such as when teams were dispatched to respond to incidents, it was necessary to reschedule appointments with the worker to complete the instrument completion.

### Analysis of results and statistics

Data were organized in Microsoft Excel^®^ and statistically analyzed using R version 4.2.0. Continuous variables were presented as dispersion using interquartile ranges and standard deviations, according to the data distribution, and as measures of central tendency using mean and median. Categorical variables were described as absolute and relative frequencies.

The relationship among MPD (SRQ ≥ 7) variables and outcome was investigated by estimating the relative risk, using a generalized estimating equations model with Poisson distribution and robust variances. Whether there was a change in the SRQ score between the two time points was assessed using multiple analysis of variance for repeated measures^(9)^. P-values<0.05 were considered significant.

## RESULTS

The sample consisted of 52 SAMU workers, who were monitored at both study moments. Of these, the largest percentage were male workers (55.8%; n=29), self-declared white (65.4%; n=34), married (75%; n=39), and with a higher education degree (80.8%; n=42). The mean age was 44.69±8.21, and the median number of children was 1.5^(1-2)^. The professional categories were nursing technicians/assistants (48.1%; n=25), nurses (23.1%; n=12), drivers (21.2%; n=11), and physicians (7.7%; n=4). Table 1 shows the distribution of SAMU workers in relation to MPD before and after two years of the COVID-19 pandemic, and associated sociodemographic, clinical, and work factors.

**Table 1:** Distribution of Mobile Emergency Care Service workers with minor psychiatric disorders in times 1 and 2 according to sociodemographic, clinical, and work data (N=52), Porto Alegre, Rio Grande do Sul, Brazil, 2019-2022

Sociodemographic, clinical, and work data	Presence of minor psychiatric disorders				*p* ^*^
	Time 1		Time 2		
	Yes (n=17)	No (n=35)	Yes (n=21)	No (n=31)	
Sociodemographic variables					
Sex					
Male	5 (17.2%)	24 (82.8%)	9 (31.0%)	20 (69.0%)	0.25
Female	12 (52.2%)	11 (47.8%)	12 (52.2%)	11 (47.8%)	
**Age**	44.01 (±8.16)		45.41 (±7.57)		0.25
**Skin color**					
White	11 (32.4%)	23 (67.6%)	12 (35.3%)	22 (64.7%)	0.25
Black, brown, and others	6 (33.3%)	12 (66.7%)	9 (50.0%)	9 (50.0%)	
**Education (years of study)**					
Elementary and high school	2 (22.2%)	7 (77.8%)	1 (11.1%)	8 (88.9%)	0.59
Higher education (> 11 years)	14 (33.3%)	28 (66.7%)	19 (45.2%)	23 (54.8%)	
**Marital status**					
Married	11 (28.2%)	28 (71.8%)	15 (38.5%)	24 (61.5%)	0.25
Single or widowed	6 (46.2%)	7 (53.8%)	6 (46.2%)	7 (53.8%)	
**Children**	1 (0±2)		1 (1±2)		0.25
**Clinical variables**					
**Smoking**					
Yes	1 (14.3%)	6 (85.7%)	2 (28.6%)	5 (71.4%)	0.25
No	16 (35.6%)	29 (64.4%)	19 (42.2%)	26 (57.8%)	
**Alcohol**					
Yes	7 (30.4%)	16 (69.6%)	9 (39.1%)	14 (60.9%)	0.30
No	10 (34.5%)	19 (65.5%)	12 (41.4%)	17 (58.6%)	
**Comorbidities**					
Yes	10 (52.6%)	9 (47.4%)	12 (63.2%)	7 (36.8%)	0.21
No	7 (21.2%)	26 (78.8%)	9 (27.3%)	24 (72.7%)	
**Use of medications**					
Yes	12 (52.2%)	11 (47.8%)	13 (56.5%)	10 (43.5%)	0.15
No	5 (17.2%)	24 (82.8%)	8 (27.6%)	21 (72.4%)	
**Weight**	73.70 (±14.69)		79.52 (±14.17)		0.23
**Abdominal circumference**	95.14 (±11.07)		95.10 (±12.27)		0.69
**Hip circumference**	106.38 (±8.54)		106.41 (±7.82)		0.64
**Sleep (in hours)**					
≤ 6 hours	14 (45.2%)	17 (54.8%)	18 (58.1%)	13 (41.9%)	0.33
> 6 hours	3 (14.3%)	18 (85.7%)	3 (14.3%)	18 (85.7%)	
**Physical health assessment**	2.9 (±1.1)		3.0 (±0.8)		0.41
**Mental health assessment**	3.3 (±1.0)		2.8 (±1.0)		0.59
**Work variables**					
**Professional category**					
Nursing technician/assistant	8 (32%)	17 (68.0%)	12 (48%)	13 (52.0%)	0.25
Driver	1 (9.1%)	10 (90.9%)	1 (9.1%)	10 (90.9%)	
Nurse	7 (58.3%)	5 (41.7%)	7 (58.3%)	5 (41.7%)	
Physician	1 (25%)	3 (75.0%)	1 (25%)	3 (75.0%)	
**Years of experience**	18.35 (±6.55)		19.90 (±6.81)		0.25
**Management position**					
Yes	5 (55.6%)	4 (44.4%)	4 (44.4%)	5 (55.6%)	0.25
No	12 (27.9%)	31 (72.1%)	17 (39.5%)	26 (60.5%)	
**Shift**					
Daytime	7 (25.9%)	20 (74.1%)	7 (25.9%)	20 (74.1%)	0.32
Nighttime	8 (40%)	12 (60.0%)	12 (60.0%)	8 (40.0%)	
Both	2 (40%)	3 (60.0%)	2 (40.0%)	3 (60.0%)	
**Works at another institution**					
Yes	4 (50%)	4 (50.0%)	4 (50%)	4 (50.0%)	0.25
No	13 (29.5%)	31 (70.5%)	17 (38.6%)	27 (61.4%)	
**Departures**					
Yes	12 (33.3%)	24 (66.7%)	15 (41.7%)	21 (58.3%)	0.59
No	5 (31.3%)	11 (68.8%)	6 (37.5%)	10 (62.5%)	
**Reason for removal**					
Musculoskeletal	3 (42.9%)	4 (57.1%)	4 (57.1%)	3 (42.9%)	0.38
Psychological	1 (50%)	1 (50.0%)	1 (50.0%)	1 (50.0%)	
Other reasons	8 (29.6%)	19 (70.4%)	10 (37.0%)	17 (63.0%)	
**Days away**	24.14 (±34.59)		36.81 (±48.09)		0.11
**Violence in the workplace**					
Yes	12 (46.2%)	14 (53.8%)	14 (53.8%)	12 (46.2%)	0.41
No	5 (19.2%)	21 (80.8%)	7 (26.9%)	19 (73.1%)	

* *Generalized estimating equations model with Poisson distribution and robust variances.*

The effect of the COVID-19 pandemic on MPD in workers working at SAMU was estimated, as shown in Table 2.

**Table 2:** Effect of the COVID-19 pandemic on minor psychiatric disorders in Mobile Emergency Care Service workers (N=52), Porto Alegre, Rio Grande do Sul, Brazil, 2019-2022

Minor psychiatric disorders				
**Time 1**	**Time 2**	**Effect size**	**Statistical power**	*p* ^*^
5.11 (±4.64)	5.78 (±5.19)	0.23	0.33	0.12

* *Multiple analysis of variance for repeated measures.*

Despite the increased means from time 1 (pre-pandemic) to time 2 (pandemic), no differences were observed between the times studied regarding the effect of the COVID-19 pandemic on MPD.

Based on the selection of sociodemographic, clinical and work data associated with MPD, the intensity of the associations among variables was examined using Poisson regression, as shown in Table 3.

**Table 3:** Sociodemographic, clinical, and occupational data associated with minor psychiatric disorders (N=52), Porto Alegre, Rio Grande do Sul, Brazil, 2019-2022

Sociodemographic, clinical and work data	RR (95%CI)	p^*^
**Sociodemographic variables** Female	2.10 (1.11 - 3.97)	**0.02**
**Clinical variables**		
Comorbidities	2.33 (1.13 - 4.08)	**0.00**
Use of ongoing medications	2.37 (1.30 - 4.33)	**0.01**
Weight	0.97 (0.95 - 0.99)	**0.02**
Sleeping six hours or less	2.81 (1.3 - 6.08)	**0.01**
Self-assessed physical health	0.64 (0.52 - 0.78)	**< 0.001**
Self-assessed mental health	0.56 (0.46 - 0.68)	**< 0.001**
**Labor variables**		
Nursing technician/assistant	4.44 (1.19 - 16.56)	**0.03**
Nurse	6.41 (1.69 - 24.24)	**0.01**

* *Generalized estimating equations model with Poisson distribution and robust variances; 95%CI - 95% Confidence Interval; RR - relative risk.*

Table 4 also explores a regression model to identify physical health and work-related variables that explain MPD.

**Table 4:** Musculoskeletal symptoms and work-related injuries associated with minor psychiatric disorders in Mobile Emergency Care Service workers (N=52), Porto Alegre, Rio Grande do Sul, Brazil, 2019-2022

	Musculoskeletal symptoms and work-related injuries	RR	*p* ^*^
**Standardised Nordic Questionnaire**	Neck pain or discomfort (past 12 months)	2.02 (1.15 - 3.54)	**0.01**
	Lower back pain or discomfort (past 12 months)	2.22 (1.24 - 3.97)	**0.01**
	Impediments due to neck problems	2.07 (1.31 - 3.28)	**<0.01**
	Impediments due to lower back problems	1.42 (0.87 - 2.30)	0.16
	Neck pain or discomfort (past seven days)	2.23 (1.42 - 3.48)	**<0.01**
	Lower back pain or discomfort (past seven days)	2.35 (1.33 - 4.15)	**<0.01**
***Escala de Avaliação dos Danos Relacionados ao Trabalho***	Physical harm	13.22 (3.11 - 56.25)	**<0.01**
	Social harm	3.59 (2.12 - 6.06)	**<0.01**
	Psychological harm	4.20 (2.21 - 7.98)	**<0.01**

* *Generalized estimating equations model with Poisson distribution and robust variances; RR - relative risk.*

Having pain or discomfort in the neck and lower back in the last 12 months increases the risk of developing MPD by 2.02 and 2.22 times, respectively. Inability to perform activities in the last 12 months due to pain or discomfort in the neck and lower back increases the risk of developing MPD by 2.07 and 1.42 times, respectively. Pain or discomfort in the lower back and neck in the last seven days increases the risk of developing MPD by 2.23 and 2.35 times, respectively. Work-related harm, social, psychological, and physical harm increase the risk of developing MPD by 3.59, 4.20, and 13.22 times, respectively.

## DISCUSSION

The prevalence of MPD was 32.7% (n=17) in time 1, pre-pandemic, and 40.4% (n=21) in time 2, two years after the pandemic, corroborating the literature, which demonstrated high prevalence in the pandemic period (49.3%^(15)^) and similar in the pre-pandemic period (32.6%^(11)^ and 32.2^(16)^) in different work contexts. Despite a 7.7% increase in MPD in the sample of monitored workers, the effect was not considered statistically significant. This result suggests that, along with the challenges of the health crisis, an increase in resilience and cooperation among teams working on the front lines was also observed. Women had a higher prevalence of MPD compared to men, as already noted in the literature^(11)^. Furthermore, women are at greater risk of mental health problems, with higher anxiety scores compared to men^(17)^.

Medication use and comorbidities have been shown to be related to a higher prevalence of MPD, an association frequently observed in chronic diseases such as diabetes, hypertension, respiratory diseases, musculoskeletal disorders, and anxiety disorders^(16)^. During the pandemic, studies identified an increase in the misuse of psychotropic medications by healthcare workers due to burnout^(18)^.

Due to the weight change associated with MPD, it is worth noting that the literature has pointed to the implications of the COVID-19 pandemic on dietary changes through emotional or excessive consumption of foods that are denser in calories and high in saturated fat^(19)^, as per a Brazilian study that showed that 78.5% of healthcare workers observed changes in their diet due to binge eating and increased carbohydrate intake combined with nocturnal habits that increase the propensity for weight gain^(20)^. Additionally, anxiety, depression, and emotional eating negatively influence pandemic-related concerns^(19)^.

Sleeping six hours or less was associated with MPD, being a stressful and detrimental factor for healthcare workers’ mental health, reflecting high rates of insomnia in the United States^(21)^. Furthermore, a Brazilian study that investigated changes in healthcare workers’ daily lives and sleep habits during the global health crisis caused by COVID-19 found that 30.3% had moderate difficulty falling asleep, and 30.3% had moderate difficulty staying asleep. Furthermore, 42.3% expressed dissatisfaction with their current sleep pattern; 40.4% were bothered by how their sleep pattern interfered with daily activities; and 34.9% were worried and stressed about sleep problems^(20)^. The heavy workload resulting from the pandemic prevented workers from having adequate rest, interfering with sleep quality and the restoration it provides. Furthermore, a Swedish study of 3,495 healthcare workers six months into the pandemic reported a significant decline in sleep quality, culminating in higher levels of depression and mental and physical fatigue^(22)^.

In this study, higher prevalences of MPD were tracked among nursing technicians and nurses, in line with other studies^(11)^. The stressors experienced by nursing technicians are related to working conditions, interpersonal interactions, lack of recognition, insufficient pay, lack of professional encouragement, and disrespect^(11)^. Furthermore, another study found that nursing technicians are more susceptible to developing traumatic stress when compared to physicians and nurses^(23)^. However, another study indicates that nurses have a higher risk of psychological impairment, more specifically depression, anxiety and post-traumatic stress disorder^(24)^.

Poorer self-rated mental and physical health are associated with MPD. A cross-sectional survey conducted by the Chinese Center for Disease Control and Prevention found that 15% of healthcare workers in China rate their self-rated health as very poor, due to long work hours and concerns about COVID-19^(25)^. Therefore, good self-rated health was significantly associated with the feeling of being sufficiently protected by available work protective equipment and prepared to deal with individuals affected by COVID-19, according to a cross-sectional survey conducted with workers in the German emergency medical service^(26)^.

Healthcare workers’ psychological health has gained prominence due to exposure to various biological, physical, chemical, ergonomic, and psychosocial risks^(11)^. With the pandemic, there was an increase in levels of depression and anxiety associated with care and contact with individuals infected with the coronavirus^(27)^. Furthermore, living with these symptoms can evolve into psychiatric disorders^(23)^, in addition to physical disabilities, supporting the findings of this study, which showed an increase in the prevalence of MPD associated with musculoskeletal symptoms.

A study of healthcare workers caring for individuals with COVID-19 in the United Arab Emirates showed that anxiety scores increase due to physical fatigue and musculoskeletal pain due to stress, burnout, fear of being infected and infecting their family members, as well as sleep disturbances and emotional impairment^(28)^. These situations lead to the occurrence of physical symptoms, with fatigue being the most reported symptom (28.4%), followed by muscle pain (14.6%) and back pain (11.9%)^(29)^.

In this study, the neck and lower back were the most affected by musculoskeletal symptoms and associated with MPD, which can be understood by ergonomic factors and leading to chronic low back pain in nurses, physicians, and drivers, due to frequent curvature and flexion of the trunk, heavy lifting and long periods of sitting, in addition to occupational stress, high psychological demand, low job satisfaction, and shift work^(7)^. Frequent exposure to these factors leads to an increased prevalence of work-related injuries, supporting the findings of this study.

The physical demands experienced by these workers, such as the use of force, repetitive movements, and inadequate postures during patient care and transportation, constitute risk factors for psychological and musculoskeletal disorders^(30-32)^. Furthermore, prior to the pandemic, there were already portraits of precarious work due to low pay and devaluation of workers, but, with the advent of the pandemic, there was a worsening of working conditions^(33)^ attributed to the excess of tasks, long working hours, staff shortages and insufficient material resources, in addition to the exacerbation of psychological and social disorders^(2)^.

### Study limitations

The potential limitations of this study are related to the high number of losses, which resulted in a reduction in the sample in the second moment (during/after the pandemic), making it impossible to analyze other impacts, in addition to the healthy worker bias.

### Contributions to nursing, health or public policy

The findings of this study contribute to understanding the work environment and factors associated with the risk of mental and physical illness among workers, with a focus on analyzing the impact of the COVID-19 pandemic. The results can support the identification and development of preventive strategies, as well as the formulation and strengthening of public policies focused on occupational health in pre-hospital settings.

## CONCLUSIONS

This study identified an increased prevalence of MPD among SAMU workers during/after the COVID-19 pandemic, although no proven effect was found. MPD was at higher risk for women, those taking medications, those with weight and sleep disorders, poor self-rated physical and mental health, and those working in nursing technicians or nurses. Furthermore, MPD was associated with musculoskeletal symptoms, particularly pain and discomfort in the neck and lower back. Regarding work and risk of illness, MPD was associated with physical, social, and psychological harm.

## Data Availability

The research data are available within the article.
