# Supplementary material for: COVID-19 pandemic in minor psychiatric disorders among mobile emergency service workers: cohort
Source: Rev Bras Enferm. 2026 May 11;79:e20250098. doi: 10.1590/0034-7167-2025-0098 (PMC13169632; doi:10.1590/0034-7167-2025-0098)
Supplement: Translation

# Translation: Pandemia de COVID-19 nos transtornos mentais menores dos trabalhadores dos serviços móveis de urgência: coorte

**DOI:** 10.1590/0034-7167-2025-0098pt

**Authors:** Natasha da Silva Indruczaki Guedes, Polla Victória Paim Rodrigues Finckler, Silviamar Camponogara, Keyla Cristiane do Nascimento, Juliana Petri Tavares, Daiane Dal Pai

**Affiliations:** I Universidade Federal do Rio Grande do Sul. Porto Alegre, Rio Grande do Sul, Brasil; II Universidade Federal de Santa Maria. Santa Maria, Rio Grande do Sul, Brasil; III Universidade Federal de Santa Catarina. Florianópolis, Santa Catarina, Brasil

**Keywords:** COVID-19, Serviços Médicos de Emergência, Saúde Ocupacional, Transtornos Mentais, Transtornos Traumáticos Cumulativos.

## Abstract

**Objetivos:**

mensurar o efeito da pandemia de COVID-19 sobre os distúrbios psíquicos menores em trabalhadores do Serviço de Atendimento Móvel de Urgência.

**Métodos:**

estudo de coorte retrospectiva e prospectiva, realizada em uma capital sul brasileira. Foram acompanhados 52 trabalhadores (poder amostral 88,2%) na pré-pandemia e após dois anos, com aplicação do *Self-Reporting Questionnaire*. Estimou-se o risco relativo pelo modelo de equações de estimações generalizadas e análise múltipla de variância para medidas repetidas, considerando-se p<0,05.

**Resultados:**

os distúrbios aumentaram de 32,7% (n=17) para 40,4% (n=21) após dois anos da pandemia (efeito 0,23; p=0,12), com risco aumentado para o sexo feminino (RR=2,10; IC95% 1,11-3,97), comorbidades (RR=2,33; IC95% 1,13-4,08), uso de medicamentos (RR=2,37; IC95% 1,30-4,33), técnico de enfermagem (RR=4,44; IC95% 1,19-16,56) e enfermeiro (RR=6,41; IC95% 1,69-24,24). Os distúrbios associaram-se à dor no pescoço e lombar, danos físicos, sociais e psicológicos (p**<**0,01).

**Conclusões:**

os distúrbios psíquicos menores apresentaram prevalência elevada, intensificando-se durante a pandemia.

## INTRODUÇÃO

A pandemia ocasionada pela COVID-19 exigiu reorganização dos componentes do sistema de saúde brasileiro. Na atenção às urgências, o Serviço de Atendimento Móvel de Urgência (SAMU) passou por mudanças em fluxos e protocolos institucionais, além da diferenciação no perfil dos atendimentos^(1)^. Ademais, houve aumento no tempo resposta das equipes, considerando a recomendação de limpeza das ambulâncias entre atendimentos e transportes de pacientes^(2)^.

O SAMU integra a Rede de Atenção às Urgências no sistema de saúde brasileiro como componente móvel pré-hospitalar, com a finalidade de coordenar o fluxo e prestar assistência imediata, efetiva e segura a vítimas acometidas por agravos à saúde de diversas naturezas. A atuação do serviço ocorre por meio do deslocamento de equipes especializadas de suporte básico e avançado, direcionadas a situações que apresentam risco à vida, demandando intervenções imediatas, e transporte rápido e seguro para os serviços de referência^(1)^.

Durante o enfrentamento da pandemia de COVID-19, trabalhadores do atendimento pré-hospitalar (APH) vivenciaram medo, insegurança, receio de contrair a doença e contaminar familiares^(1)^, entre outros sentimentos que impactaram suas vivências profissionais e rotina laboral. O período pandêmico exacerbou características pré-existentes em rotinas laborais do APH. Inúmeros fatores podem estar relacionados ao desenvolvimento do sofrimento psíquico desses trabalhadores, como a falta de segurança no ambiente de trabalho, ausência de materiais destinados à proteção individual do trabalhador e excesso de horas de trabalho^(3)^.

A presença de fatores estressantes relacionados aos atendimentos de emergência em equipe foi descrita na literatura, sendo destacadas situações de insônia, depressão e ansiedade, além de estresse e transtorno pós-traumático^(4,5)^. Estudo iraniano constatou que a atuação no resgate dos indivíduos acometidos pela COVID-19 e vivências de sofrimento aumentaram os níveis de ansiedade, levando ao estresse psicológico no ambiente de trabalho diante das preocupações com a infecção, o medo de propagação da doença, o sofrimento dos pacientes e familiares, e os danos causados pelos equipamentos de proteção individual nos enfermeiros^(6)^. Estudos internacionais identificaram maior probabilidade de acometimento psíquico nos trabalhadores da saúde que atuaram na linha de frente como depressão, ansiedade, insônia e angústia^(7,8)^.

No Brasil, não foram identificados estudos que avaliaram os impactos à saúde psíquica dos trabalhadores do SAMU antes e após o surgimento da pandemia ocasionada pela COVID-19, o que justifica a realização do estudo, considerando que os impactos de crise sanitária devem ser mensurados em todas as esferas de atenção à saúde.

### Relevância do estudo

Este artigo é produto de dissertação intitulada “Impacto da pandemia da Covid-19 no trabalho e adoecimento dos profissionais do Serviço de Atendimento Móvel de Urgência”^(9)^, depositada no repositório institucional (http://hdl.handle.net/10183/259608).

## OBJETIVOS

Mensurar o efeito da pandemia de COVID-19 sobre os distúrbios psíquicos menores (DPM) em trabalhadores do SAMU.

## MÉTODOS

### Aspectos éticos

Foram respeitadas as diretrizes éticas nacionais que regem a pesquisa em saúde, ciências humanas e sociais, conforme as Resoluções n^o^ 466/2012 e n^o^ 510/2016 do Conselho Nacional de Saúde. Todos os participantes consentiram mediante assinatura do Termo de Consentimento Livre e Esclarecido.

### Desenho, período e local do estudo

Trata-se de estudo de coorte retrospectiva e prospectiva, de abordagem quantitativa, conforme as diretrizes do *STrengthening the Reporting of OBservational studies in Epidemiology*.

Os dados foram coletados em períodos diferentes, com coletas de dezembro de 2019 a fevereiro de 2020 (pré-pandemia) e após dois anos em março de 2022 (durante a pandemia).

A pesquisa foi conduzida no SAMU de Porto Alegre, no estado do Rio Grande do Sul, Brasil. O SAMU faz a cobertura de 1.332.845 milhões de habitantes e um território de 495.390 km^2(10)^, por intermédio de 16 equipes, sendo que três são destinadas ao Suporte Avançado de Vida, enquanto as demais, contendo com 13 unidades, prestam Suporte Básico de Vida.

### População ou amostra; critérios de inclusão e exclusão

A população foi composta por 260 trabalhadores de saúde (enfermeiros, condutores, médicos e técnicos de enfermagem) que atuavam na assistência às urgências.

A amostra pré-pandemia foi composta por 113 trabalhadores do SAMU. No segundo momento de coleta, realizado dois anos após a pandemia, os trabalhadores que haviam participado do primeiro momento foram novamente incluídos. A amostra final contou com 52 participantes, enquanto 61 foram perdidos devido afastamentos e óbitos.

O poder para testar a diferença mínima de 2 pontos na média das diferenças do *Self-Reporting Questionnaire* (SRQ-20) entre os grupos pré e pós é de 88,2%, considerando o nível de significância de 5%, tamanho amostral igual a 52 indivíduos e desvio padrão das diferenças igual a 4,5 pontos^(11)^. Foi utilizada a ferramenta *PSS Health* na versão *online*.

Todos os trabalhadores foram convidados a integrar a pesquisa, sendo selecionados por conveniência, considerando que estavam trabalhando durante os períodos de coleta de dados.

Foram incluídos os trabalhadores que atuam em atividades assistenciais no serviço e vínculo mínimo de dois anos. Foram excluídos aqueles que estavam de férias ou afastados durante a coleta de dados. No segundo momento do estudo, realizado dois anos após a pandemia, incluiu-se os trabalhadores que se mantiveram na assistência e que haviam respondido aos instrumentos no primeiro momento do estudo.

### Protocolo do estudo

A coleta de dados ocorreu por instrumentos contendo dados sociodemográficos, clínicos e laborais, e o SRQ-20. Para obtenção dos dados clínicos, além de questões gerais sobre a saúde, foi aplicado o *Standardised Nordic Questionnaire* (SNQ). Para obtenção dos dados laborais, foi aplicada a Escala de Avaliação dos Danos Relacionados ao Trabalho (EADRT).

Os DPM foram rastreados por meio do SRQ-20. Esse instrumento conta com 20 questões, que pontuam de 0 ou 1, e rastreiam sintomas não psicóticos, como irritabilidade, lapsos de memória, insônia, dificuldade de concentração e fadiga, sem configurar um diagnóstico clínico. Possuir sete respostas positivas ou mais foi considerado desfecho positivo^(12)^. O SNQ analisa os sintomas musculoesqueléticos na saúde ocupacional^(13)^.

A EADRT é uma das escalas que compõe o Inventário do Trabalho e Risco de Adoecimento, avaliando danos laborais à sua saúde na opinião do trabalhador. A cada questão é atribuída uma pontuação que varia de 1 a 6. Escore inferior a 1,9 pontos significa uma percepção mais positiva dos danos, considerando-os toleráveis; pontuações entre 2 e 3 refletem uma avaliação moderada a crítica; pontuações de 3,1 e 4 sinalizam impactos de moderados para frequentes, sendo considerados graves; enquanto escores acima de 4,1 apontam uma avaliação mais negativa, associada a doenças ocupacionais^(14)^. Neste estudo, para maximizar as diferenças entre os grupos, as classificações foram reduzidas em duas: com doença ocupacional (escore ≥ 4,1); e com situações graves relacionadas ao trabalho (escore ˂ 4).

Os dados foram coletados nas bases do SAMU, conforme horário e disponibilidade do trabalhador, que foram convidados presencialmente para participação do estudo em ambas fases. Os instrumentos foram aplicados por uma equipe de pesquisa treinada. Em algumas situações, como o acionamento das equipes para atendimento de ocorrências, houve a necessidade de remarcação juntamente com o trabalhador para finalização do preenchimento dos instrumentos.

### Análise dos resultados e estatística

Os dados foram organizados no *Microsoft Excel^®^* e submetidos à análise estatística utilizando o *software* R, versão 4.2.0. As variáveis contínuas foram apresentadas por dispersão através de intervalos interquartílicos e desvio padrão, de acordo com a distribuição dos dados, e por medidas de tendência central, através de média e mediana. Já as variáveis categorias foram descritas por frequências absolutas e relativas.

A relação entre as variáveis e o desfecho dos DPM (SRQ ≥ 7) foi investigada por meio da estimativa do risco relativo, utilizando-se um modelo de equações de estimações generalizadas com distribuição Poisson e variâncias robustas. Foi avaliado se houve alteração no escore do SRQ entre os dois momentos com análise múltipla de variância para medidas repetidas^(9)^. Consideraram-se significativos os valores de p<0,05.

## RESULTADOS

A amostra foi composta por 52 trabalhadores do SAMU, sendo acompanhada nos dois momentos do estudo. Desses, o maior percentual compreendeu trabalhadores do sexo masculino (55,8%; n=29), autodeclarados de cor branca (65,4%; n=34), casados (75%; n=39) e com ensino superior completo (80,8%; n=42). A média de idade foi de 44,69±8,21, e a mediana de filhos foi de 1,5^(1-2)^. As categorias profissionais incluíram técnicos/auxiliares de enfermagem (48,1%; n=25), enfermeiros (23,1%; n=12), condutores (21,2%; n=11) e médicos (7,7%; n=4). Na Tabela 1, estão distribuídos os trabalhadores do SAMU em relação aos DPM antes e após dois anos da pandemia de COVID-19, e os fatores sociodemográficos, clínicos e laborais associados.

**Tabela 1:** Distribuição dos trabalhadores do Serviço de Atendimento Móvel de Urgência com presença de distúrbios psíquicos menores nos tempos 1 e 2 segundo dados sociodemográficos, clínicos e laborais (N=52), Porto Alegre, Rio Grande do Sul, Brasil, 2019-2022

Dados sociodemográficos, clínicos e laborais	Presença de distúrbios psíquicos menores				*p* ^*^
	Tempo 1		Tempo 2		
	Sim (n=17)	Não (n=35)	Sim (n=21)	Não (n=31)	
Variáveis sociodemográficas					
Sexo					
Masculino	5 (17,2%)	24 (82,8%)	9 (31,0%)	20 (69,0%)	0,25
Feminino	12 (52,2%)	11 (47,8%)	12 (52,2%)	11 (47,8%)	
**Idade**	44,01 (±8,16)		45,41 (±7,57)		0,25
**Cor da pele**					
Branca	11 (32,4%)	23 (67,6%)	12 (35,3%)	22 (64,7%)	0,25
Negra, parda e outras	6 (33,3%)	12 (66,7%)	9 (50,0%)	9 (50,0%)	
**Escolaridade (anos de estudo)**					
Ensino fundamental e médio	2 (22,2%)	7 (77,8%)	1 (11,1%)	8 (88,9%)	0,59
Ensino superior (> 11 anos)	14 (33,3%)	28 (66,7%)	19 (45,2%)	23 (54,8%)	
**Situação conjugal**					
Casado(a)	11 (28,2%)	28 (71,8%)	15 (38,5%)	24 (61,5%)	0,25
Solteiro(a) ou viúvo(a)	6 (46,2%)	7 (53,8%)	6 (46,2%)	7 (53,8%)	
**Filhos**	1 (0±2)		1 (1±2)		0,25
**Variáveis clínicas**					
**Tabagismo**					
Sim	1 (14,3%)	6 (85,7%)	2 (28,6%)	5 (71,4%)	0,25
Não	16 (35,6%)	29 (64,4%)	19 (42,2%)	26 (57,8%)	
**Álcool**					
Sim	7 (30,4%)	16 (69,6%)	9 (39,1%)	14 (60,9%)	0,30
Não	10 (34,5%)	19 (65,5%)	12 (41,4%)	17 (58,6%)	
**Comorbidades**					
Sim	10 (52,6%)	9 (47,4%)	12 (63,2%)	7 (36,8%)	0,21
Não	7 (21,2%)	26 (78,8%)	9 (27,3%)	24 (72,7%)	
**Uso de medicações**					
Sim	12 (52,2%)	11 (47,8%)	13 (56,5%)	10 (43,5%)	0,15
Não	5 (17,2%)	24 (82,8%)	8 (27,6%)	21 (72,4%)	
**Peso**	73,70 (±14,69)		79,52 (±14,17)		0,23
**Circunferência abdominal**	95,14 (±11,07)		95,10 (±12,27)		0,69
**Circunferência quadril**	106,38 (±8,54)		106,41 (±7,82)		0,64
**Sono (em horas)**					
≤ 6 horas	14 (45,2%)	17 (54,8%)	18 (58,1%)	13 (41,9%)	0,33
> 6 horas	3 (14,3%)	18 (85,7%)	3 (14,3%)	18 (85,7%)	
**Avaliação da saúde física**	2,9 (±1,1)		3,0 (±0,8)		0,41
**Avaliação da saúde mental**	3,3 (±1,0)		2,8 (±1,0)		0,59
**Variáveis laborais**					
**Categoria profissional**					
Técnico(a)/auxiliar de enfermagem	8 (32%)	17 (68,0%)	12 (48%)	13 (52,0%)	0,25
Condutor(a)	1 (9,1%)	10 (90,9%)	1 (9,1%)	10 (90,9%)	
Enfermeiro(a)	7 (58,3%)	5 (41,7%)	7 (58,3%)	5 (41,7%)	
Médico(a)	1 (25%)	3 (75,0%)	1 (25%)	3 (75,0%)	
**Anos de experiência**	18,35 (±6,55)		19,90 (±6,81)		0,25
**Cargo de chefia**					
Sim	5 (55,6%)	4 (44,4%)	4 (44,4%)	5 (55,6%)	0,25
Não	12 (27,9%)	31 (72,1%)	17 (39,5%)	26 (60,5%)	
**Turno**					
Diurno	7 (25,9%)	20 (74,1%)	7 (25,9%)	20 (74,1%)	0,32
Noturno	8 (40%)	12 (60,0%)	12 (60,0%)	8 (40,0%)	
Ambos	2 (40%)	3 (60,0%)	2 (40,0%)	3 (60,0%)	
**Trabalha em outra instituição**					
Sim	4 (50%)	4 (50,0%)	4 (50%)	4 (50,0%)	0,25
Não	13 (29,5%)	31 (70,5%)	17 (38,6%)	27 (61,4%)	
**Afastamentos**					
Sim	12 (33,3%)	24 (66,7%)	15 (41,7%)	21 (58,3%)	0,59
Não	5 (31,3%)	11 (68,8%)	6 (37,5%)	10 (62,5%)	
**Motivo do afastamento**					
Osteomusculares	3 (42,9%)	4 (57,1%)	4 (57,1%)	3 (42,9%)	0,38
Psicológicos	1 (50%)	1 (50,0%)	1 (50,0%)	1 (50,0%)	
Outros motivos	8 (29,6%)	19 (70,4%)	10 (37,0%)	17 (63,0%)	
**Dias de afastamento**	24,14 (±34,59)		36,81 (±48,09)		0,11
**Violência no ambiente de trabalho**					
Sim	12 (46,2%)	14 (53,8%)	14 (53,8%)	12 (46,2%)	0,41
Não	5 (19,2%)	21 (80,8%)	7 (26,9%)	19 (73,1%)	

* *Modelo de equações de estimações generalizadas com distribuição Poisson e variâncias robustas.*

Estimou-se o efeito da pandemia de COVID-19 nos DPM em trabalhadores que atuam no SAMU, conforme a Tabela 2.

**Tabela 2:** Efeito da pandemia de COVID-19 nos distúrbios psíquicos menores em trabalhadores do Serviço de Atendimento Móvel de Urgência (N=52), Porto Alegre, Rio Grande do Sul, Brasil, 2019-2022

Distúrbios psíquicos menores				
**Tempo 1**	**Tempo 2**	**Tamanho** **do efeito**	**Poder estatístico**	*p* ^*^
5,11 (±4,64)	5,78 (±5,19)	0,23	0,33	0,12

* *Análise múltipla de variância para medidas repetidas.*

Apesar dos aumentos das médias do tempo 1 (pré pandemia) para o tempo 2 (pandemia), não foram observadas diferenças entre os tempos estudados quanto ao efeito da pandemia de COVID-19 nos DPM.

A partir da seleção dos dados sociodemográficos, clínicos e laborais associados aos DPM, a intensidade das associações entre as variáveis foi examinada utilizando regressão de Poisson, conforme a Tabela 3.

**Tabela 3:** Dados sociodemográficos, clínicos e laborais associados a distúrbios psíquicos menores (N=52), Porto Alegre, Rio Grande do Sul, Brasil, 2019-2022

Dados sociodemográficos, clínicos e laborais	RR (IC95%)	p^*^
**Variáveis sociodemográficas** Feminino	2,10 (1,11 - 3,97)	**0,02**
**Variáveis clínicas**		
Comorbidades	2,33 (1,13 - 4,08)	**0,00**
Uso de medicações contínuas	2,37 (1,30 - 4,33)	**0,01**
Peso	0,97 (0,95 - 0,99)	**0,02**
Dormir seis horas ou menos	2,81 (1,3 - 6,08)	**0,01**
Autoavaliação da saúde física	0,64 (0,52 - 0,78)	**< 0,001**
Autoavaliação da saúde mental	0,56 (0,46 - 0,68)	**< 0,001**
**Variáveis laborais**		
Técnico(a)/auxiliar de enfermagem	4,44 (1,19 - 16,56)	**0,03**
Enfermeiro(a)	6,41 (1,69 - 24,24)	**0,01**

* *Modelo de equações de estimações generalizadas com distribuição Poisson e variâncias robustas; IC95% - Intervalo de Confiança de 95%; RR - risco relativo.*

A Tabela 4 também explora um modelo de regressão, a fim de identificar variáveis de saúde física e relacionadas ao trabalho que explicam os DPM.

**Tabela 4:** Sintomas musculoesqueléticos e danos relacionados ao trabalho associados aos distúrbios psíquicos menores nos trabalhadores do Serviço de Atendimento Móvel de Urgência (N=52), Porto Alegre, Rio Grande do Sul, Brasil, 2019-2022

	Sintomas musculoesqueléticos e danos relacionados ao trabalho	RR	*p* ^*^
***Standardised Nordic Questionnaire***	Dor ou desconforto pescoço (últimos 12 meses)	2,02 (1,15 - 3,54)	**0,01**
	Dor ou desconforto lombar (últimos 12 meses)	2,22 (1,24 - 3,97)	**0,01**
	Impedimentos por problemas no pescoço	2,07 (1,31 - 3,28)	**<0,01**
	Impedimentos por problemas na lombar	1,42 (0,87 - 2,30)	0,16
	Dor ou desconforto pescoço (últimos sete dias)	2,23 (1,42 - 3,48)	**<0,01**
	Dor ou desconforto lombar (últimos sete dias)	2,35 (1,33 - 4,15)	**<0,01**
**Escala de Avaliação dos Danos Relacionados ao Trabalho**	Danos físicos	13,22 (3,11 - 56,25)	**<0,01**
	Danos sociais	3,59 (2,12 - 6,06)	**<0,01**
	Danos psicológicos	4,20 (2,21 - 7,98)	**<0,01**

* *Modelo de equações de estimações generalizadas com distribuição Poisson e variâncias robustas; RR - risco relativo.*

Apresentar dor ou desconforto no pescoço e na região lombar nos últimos 12 meses aumenta em 2,02 e 2,22 vezes, respectivamente, o risco de desenvolver DPM. O impedimento de realização de atividades nos últimos 12 meses em decorrência de dor ou desconforto no pescoço e na lombar aumenta em 2,07 e 1,42 vezes, respectivamente, o risco de desenvolver DPM. Já a dor ou desconforto na lombar e no pescoço nos últimos sete dias aumenta em 2,23 e 2,35 vezes, respectivamente, o risco de desenvolver DPM. Com relação aos danos relacionados ao trabalho, os danos sociais, psicológicos e físicos aumentam em 3,59, 4,20 e 13,22 vezes, respectivamente, o risco de desenvolver DPM.

## DISCUSSÃO

A prevalência de DPM foi de 32,7% (n=17) no tempo 1, pré-pandemia, e 40,4% (n=21) no tempo 2, após dois anos da pandemia, corroborando a literatura, que demonstrou prevalências altas no período pandêmico (49,3%^(15)^) e similares no período pré-pandêmico (32,6%^(11)^ e 32,2^(16)^) em diferentes contextos laborais. Apesar do aumento de 7,7% no DPM na amostra de trabalhadores monitorados, o efeito não foi considerado estatisticamente significativo, resultado que leva a ponderar que, juntamente com os desafios da crise sanitária, também foi possível observar o incremento de atributos de resiliência e cooperação entre as equipes que tiveram que atuar na linha de frente. As mulheres apresentaram maiores prevalências de DPM quando comparadas aos homens, conforme já apontado na literatura^(11)^. Ainda, as mulheres apresentam maior risco de acometimento da saúde mental, com escores de ansiedade maiores quando comparadas aos homens^(17)^.

O uso de medicamentos e comorbidades se mostraram relacionados a uma maior prevalência de DPM, associação frequentemente observada em doenças crônicas como diabetes, hipertensão, doenças respiratórias, distúrbios osteomusculares e transtornos de ansiedade^(16)^. Durante a pandemia, estudos identificaram o aumento na utilização indevida de medicamentos psicotrópicos por trabalhadores da saúde devido ao esgotamento^(18)^.

Devido à alteração de peso associada aos DPM, cabe sinalizar que a literatura tem apontado para as implicações da pandemia de COVID-19 nas mudanças alimentares através do consumo emocional ou excessivo de alimentos mais densos em calorias e com alto teor de gordura saturada^(19)^, conforme estudo brasileiro que evidenciou que 78,5% dos trabalhadores de saúde observaram mudanças na alimentação diante da compulsão alimentar e aumento da ingestão de carboidratos aliado aos hábitos noturnos que aumentam a propensão ao ganho de peso^(20)^. Além disso, a ansiedade, depressão e alimentação emocional influenciam negativamente as preocupações relacionadas à pandemia^(19)^.

Dormir seis horas ou menos esteve associado aos DPM, sendo fator estressante e prejudicial para saúde mental dos trabalhadores de saúde, refletindo elevados percentuais de insônia nos Estados Unidos^(21)^. Ainda, estudo brasileiro que investigou as mudanças no cotidiano e nos hábitos de sono dos trabalhadores de saúde durante a crise sanitária global ocasionada pela COVID-19 evidenciou 30,3% com dificuldade moderada para adormecer e 30,3% com dificuldade média para manter o sono. Além disso, 42,3% apresentaram insatisfação com o padrão de sono atual; 40,4% estavam incomodados em como o padrão do sono interfere nas atividades diárias; e 34,9% estavam preocupados e estressados com os problemas do sono^(20)^. A elevada carga de trabalho decorrente da pandemia impossibilitou o descanso adequado dos trabalhadores, interferindo na qualidade do sono e na restauração que o mesmo proporciona. Ademais, estudo sueco realizado com 3.495 trabalhadores de saúde após seis meses de início da pandemia relatou piora significativa da qualidade do sono, culminando em maiores níveis de depressão e fadiga mental e física^(22)^.

Neste estudo, foram rastreadas maiores prevalências de DPM entre técnicos de enfermagem e enfermeiros, indo ao encontro de outros estudos^(11)^. Os fatores estressantes vivenciados pelos técnicos de enfermagem estão relacionados às condições laborais, interações interpessoais, ausência de reconhecimento, remuneração insuficiente, falta de estímulo profissional e desrespeito^(11)^. Além disso, outro estudo verificou que técnicos de enfermagem são mais suscetíveis ao desenvolvimento de estresse traumático quando comparados com médicos e enfermeiros^(23)^. Todavia, outro estudo aponta que o enfermeiro possui um risco maior de comprometimento psicológico, mais especificamente a depressão, ansiedade e transtorno de estresse pós-traumático^(24)^.

As piores autoavaliações de saúde mental e física estão associadas aos DPM. Pesquisa transversal conduzida pelo Centro Chinês de Controle e Prevenção de Doenças evidenciou que 15% dos trabalhadores de saúde da China classificam sua autoavaliação de sua saúde como muito ruim, em decorrência das longas jornadas de trabalho e preocupações com a COVID-19^(25)^. Diante disso, a boa autoavaliação de saúde foi significativamente associada à sensação de estar suficientemente protegido pelos equipamentos de proteção do trabalho disponíveis e preparados para lidar com indivíduos acometidos pela COVID-19, conforme pesquisa transversal realizada com trabalhadores do serviço médico de emergência da Alemanha^(26)^.

O psicológico dos trabalhadores de saúde tem ganhado destaque em decorrência da exposição a diversos riscos biológicos, físicos, químicos, ergonômicos e psicossociais^(11)^. Com a pandemia, observou-se aumento dos níveis de depressão e ansiedade associados ao atendimento e contato com indivíduos infectados pelo coronavírus^(27)^. Ademais, a convivência com esses sintomas pode evoluir para transtornos mentais^(23)^, além de incapacidades físicas, corroborando os achados deste estudo, que evidenciou aumento da prevalência de DPM associada aos sintomas musculoesqueléticos.

Estudo realizado com os trabalhadores de saúde envolvidos nos cuidados dos indivíduos com COVID-19 nos Emirados Árabes Unidos demonstrou que os escores de ansiedade aumentam diante da fadiga física e dores musculoesqueléticas devido ao estresse, esgotamento, medo de serem infectados e infectares seus familiares, além dos distúrbios do sono e comprometimento emocional^(28)^. Essas situações levam a ocorrência de sintomas físicos, sendo a fadiga o sintoma mais relatado (28,4%), seguido de dor muscular (14,6%) e dor nas costas (11,9%)^(29)^.

Neste estudo, o pescoço e a região lombar foram os mais acometidos por sintomas musculoesqueléticos e associados aos DPM, podendo ser compreendidos por fatores ergonômicos e levando à lombalgia crônica em enfermeiras, médicos e condutores, diante da curvatura e flexão frequente do tronco, levantamento de peso e longos períodos sentados, além do estresse ocupacional, alta demanda psicológica, baixa satisfação profissional e trabalho por turno^(7)^. A exposição frequente a esses fatores leva ao aumento da prevalência de danos relacionados ao trabalho, corroborando os achados deste estudo.

As demandas físicas experimentadas por esses trabalhadores, como uso de força, movimentos repetitivos e posturas inadequadas durante o atendimento e transporte dos pacientes, configuram-se como fatores de risco para distúrbios psicológicos e musculoesqueléticos^(30-32)^. Além disso, previamente à pandemia, já havia retratos da precarização do trabalho perante a baixa remuneração e desvalorização dos trabalhadores, mas, com o advento da pandemia, houve o agravamento das condições de trabalho^(33)^ atribuído ao excesso de tarefas, extensas jornadas laborais, escassez de pessoal e insuficiência de recursos materiais, além da exacerbação das perturbações psicológicas e sociais^(2)^.

### Limitações do estudo

As limitações potenciais deste estudo estão relacionadas ao elevado número de perdas, que resultou na redução da amostra no segundo momento (durante/após a pandemia), impossibilitando a análise de outros impactos, além do viés do trabalhador saudável.

### Contribuições para a área da enfermagem, saúde ou políticas públicas

Os achados deste estudo contribuem para a compreensão do ambiente de trabalho e dos fatores associados ao risco de adoecimento mental e físico dos trabalhadores, com foco na análise do efeito da pandemia de COVID-19. Os resultados podem subsidiar a identificação e elaboração de estratégias preventivas, bem como apoiar a formulação e o fortalecimento de políticas públicas voltada à saúde ocupacional no contexto pré-hospitalar.

## CONCLUSÕES

Este estudo identificou o aumento da prevalência de DPM nos trabalhadores do SAMU durante/após a pandemia de COVID-19, embora sem efeito comprovado. Os DPM apresentaram maior risco para o sexo feminino, em uso de medicamentos, com alterações de peso e sono, péssima autoavaliação de saúde física e mental, além das categorias profissionais de técnico de enfermagem ou enfermeiro. Além disso, os DPM estiveram associados aos sintomas musculoesqueléticos, principalmente à dor e desconforto no pescoço e região lombar. Com relação ao trabalho e risco de adoecimento, os DPM se associaram aos danos físicos, sociais e psicológicos.

## Data Availability

Os dados de pesquisa estão disponíveis no corpo do artigo.
